# Thiocolchicoside: A Focused Review of Its Pharmacological, Therapeutic, and Toxicological Profile

**DOI:** 10.7759/cureus.109774

**Published:** 2026-05-27

**Authors:** Akash V Devi, Chitra Khanwelkar, Amol Shete

**Affiliations:** 1 Department of Pharmacology, Krishna Institute of Medical Sciences (Deemed to be University), Karad, IND; 2 Department of Pharmaceutics, Krishna Institute of Medical Sciences (Deemed to be University), Karad, IND

**Keywords:** anticancer, antimicrobial, drug repurposing, gaba agonist, muscle relaxant, nanogel, pharmacology, review article, safety profile, thiocolchicoside

## Abstract

Thiocolchicoside is a colchicoside compound that is partly synthetic and is most known for its characteristics of centrally acting muscle relaxants. It has been widely used in the management of musculoskeletal disorders due to its ability to modulate inhibitory neurotransmission. Recent research has shown potential in additional therapeutic domains, such as antimicrobial, anticancer, and anti-inflammatory. The objective of this review is to provide a comprehensive overview of the chemical profile of thiocolchicoside, including how it works, pharmacokinetics, traditional and novel therapeutic uses, safety concerns, and recent advances in drug delivery systems. Special emphasis is given to its emerging roles in oncology and infectious disease management. Relevant literature was collected from PubMed, Scopus, Google Scholar, and other scientific databases. Preclinical and clinical studies, review articles, case reports, and regulatory documents related to thiocolchicoside were reviewed to extract updated and critical information. Thiocolchicoside exerts its effects by acting as a competitive antagonist on gamma-aminobutyric acid type A (GABA-A) and glycine receptors, leading to muscle relaxation without strong sedation. Several preclinical studies suggest anti-inflammatory, analgesic, antimicrobial, and cytotoxic potential. Recent innovations include nanogel-based formulations and other targeted delivery systems to enhance bioavailability and reduce systemic toxicity. However, concerns remain regarding its genotoxicity and myelosuppressive effects, particularly at higher doses or with prolonged use. Thiocolchicoside remains a valuable agent in pain and spasm management, but its expanding therapeutic spectrum suggests opportunities for drug repurposing, especially in oncology and infectious disease. Future research ought to concentrate on safety optimization, chronic toxicity studies, and the development of novel delivery platforms to maximize therapeutic efficacy while minimizing risks.

## Introduction and background

**Figure 1:**
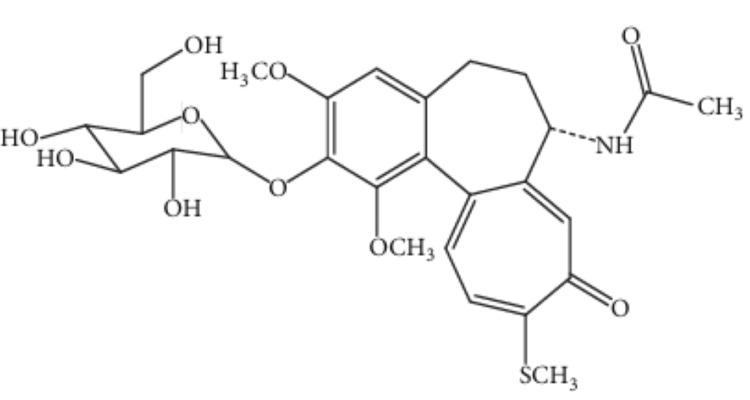
Chemical structure of Thiocolchicoside

Colchicine is a naturally occurring alkaloid isolated from the seeds of *Gloriosa superba.* *G. superba* is a member of the Liliaceae family. Its semi-synthetic derivative, Thiocolchicoside, appears as a pale yellow powder [1-3]. Thiocolchicoside is a recognized pharmaceutical agent in clinical practice, principally utilized as a central neuromuscular relaxant to alleviate pain and inflammation. In recent years, its therapeutic range has broadened beyond musculoskeletal problems. Evidence indicates that thiocolchicoside may be repurposed as an antibacterial, anticancer, and myelosuppressive drug [4-7]. The myelosuppressive effect is specifically linked to its parent chemical, colchicine [8]. Repurposing thiocolchicoside presents benefits, since clinical studies may be expedited, more cost-effective, and safer due to its established safety profile. The capacity of its metabolites to selectively target specific proteins substantiates these innovative therapeutic applications, while potentially diminishing toxicity and curtailing drug resistance. The European Medicines Agency (EMA) restricts thiocolchicoside to low-dose, short-term usage over concerns about genotoxicity and effects on fertility [9]. To mitigate these challenges, innovative medication delivery strategies, including targeted formulations, have been investigated to reduce unwanted effects. Although thiocolchicoside is commonly utilized in treating musculoskeletal disorders, its exact mechanism of action is not fully elucidated. This review aims to elucidate current evidence concerning its pharmacodynamic features, therapeutic uses, and possibilities for repurposing in oncology and infectious disease treatment. Thiocolchicoside is a semisynthetic derivative of colchicoside, incorporating sulfur and a glucoside molecule, which is naturally found in *G. superba*. These molecules are classified as phenolic glycosides (PGs), comprising sugar units, such as L-fructose, D-glucose, and L-rhamnose, linked to a phenolic framework. The IUPAC name for thiocolchicoside is N-[(10S)-3,4-dimethoxy-14-(methylsulfanyl)-13-oxo-5-{[(2S,3R,4S,5S,6R)-3,4,5-trihydroxy-6-(hydroxymethyl)oxan-2-yl]oxy}tricyclo[9.5.0.0²,⁷]hexadeca-1(16),2,4,6,11,14-hexaen-10-yl]acetamide. The molecular weight is 563.6 g/mol, and the chemical formula is C_27_H_33_NO_10_S [10-11] (Figure 1).

## Review

Materials and methods

We performed a systematic literature search utilizing PubMed, Scopus, and Google Scholar from January 2000 to March 2026. Keywords comprised “Thiocolchicoside”, “colchicine derivatives”, “muscle relaxant”, “NF-κB inhibition”, “anticancer”, “antimicrobial”, and “toxicity”. Boolean operators were utilized (e.g., Thiocolchicoside AND anticancer, Thiocolchicoside OR colchicine derivative).

Inclusion Criteria

We included peer-reviewed publications (original research, reviews, and case studies) and research documenting pharmacological, therapeutic, or toxicological information regarding Thiocolchicoside. Both preclinical (in vitro, in vivo) and clinical investigations were considered eligible for the review.

Exclusion Criteria

We excluded publications not available in English, abstracts devoid of complete text, and research not explicitly associated with Thiocolchicoside or its derivatives.

Study Selection

A total of 142 articles were recognized. Following the examination of titles and abstracts, 58 papers were removed due to irrelevance, resulting in 84 studies selected for full-text review. Among them, 52 were preclinical studies (cell culture and animal models), and 32 were clinical studies (human trials and case reports).

Data Extraction

Data were obtained about chemistry, pharmacokinetics, pharmacodynamics, therapeutic uses, toxicity, and innovative delivery systems. Evidence was classified as preclinical or clinical to emphasize translational significance.

The taxonomy is presented in Table 1.

**Table 1:** Taxonomy of Thiocolchicoside

Description	This substance is called a phenolic glycosides (PGs). These are chemicals that have a phenolic structure connected to a glycosyl molecule. A few phenolic structures are flavonoids and lignans. Natural glycosides are made up of sugar units like L-rhamnose, D-glucose, and L-fructose.
Super class	Organic oxygen compounds
Class	Organo-oxygen compounds
Sub class	Carbohydrates and their conjugates
Direct parent	Phenolic glycosides (PGs)
Alternative parents	Hexoses/O-glycosyl compounds/tropones/anisole/alkyl arylethers/alkylarylthioethers/oxanes/acetamides/secondary carboxylic acid amides/secondary alcohols
Substituents	Acetal/acetamide/alcohol/alkylarylthioether/anisole/aromatic heteropoly cyclic compound/aryl thioether/benzenoid/carbonyl group/carboxamide group
Molecular framework	Aromatic heteropoly cyclic compounds

Extraction

Thiocolchicoside is a partially generated form of colchicine and is a naturally occurring glycoside that can be found in *G. superba*. Thiocolchicoside is a semi-synthetic derivative of colchicoside, a natural glycoside isolated from the seeds of the *G. superba* plant. According to the high-performance liquid chromatography (HPLC) method, the seeds of *G. superba* contain the highest concentration of colchicine. Then, 25 mL of petroleum ether was used to remove 0.5 g of powdered plant extract. Petroleum ether was used for extraction twice, with regular shaking applied for one hour before filtration was completed. After being allowed to dry naturally, the solid precipitates were extracted using 10 milliliters of dichloromethane at room temperature for 30 minutes while being constantly shaken [12].

Synthesis

Chemical Method

**Figure 2:**
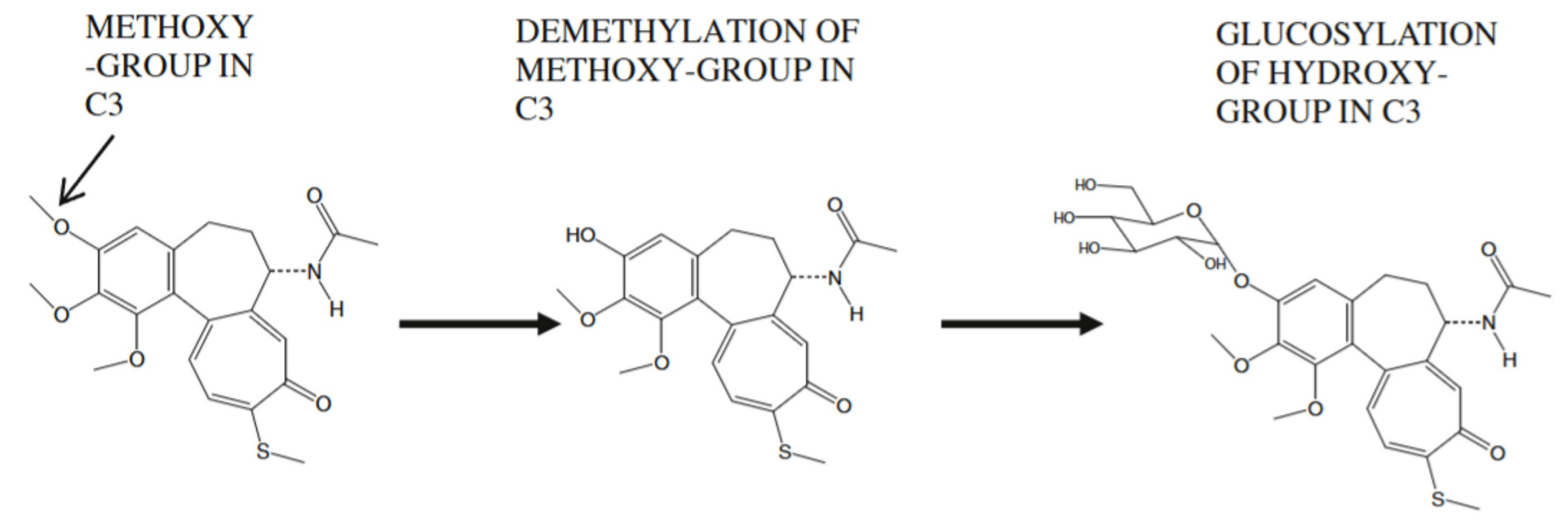
Biotransformation of Thiocolchicine (left) to Thiocolchicoside (right)

Colchicine is a precursor of Thiocolchicoside. Colchicoside, which is derived from Colchicaceae seeds, can be thiomethylated to produce Thiocolchicoside. It is produced through the glycosylation of thiocolchicine, which involves the reaction of demethyl-thiocolchicine with 2,3,4,6-tetra-O-acetyl-α-D-glucopyranosyl bromide. At first, thiomethylation transforms colchicine into the more stable thiocolchicine. Chemical techniques can be used to convert the latter into Thiocolchicoside, which involves a series of subsequent steps. The effective implementation of this approach necessitates previous demethylation at the C‑3 site, and then it must be glycosylated at the same point. However, conversion rates are low because chemical demethylation is not selective in certain regions. This leads to a mix of different 1-,2-,3-demethyl derivatives and didemethyl derivatives. In addition, the majority of the reagents utilized for the two reactions - glucosylation and demethylation - are costly and hazardous, and the subsequent process requires complex and expensive downstream activities [12] (Figure *2*).

Microbial Method

It was found that mutated *Bacillus megaterium* could produce Thiocolchicoside by doubling the rate of biotransformation of thiocolchicine, so pilot studies for the selection of the best producing strains were performed in 300 mL Erlenmeyer flasks, containing 50 mL of SF2 medium, at 30 °C on a rotary shaker at 250 rpm for four days. Colchicine and thiocolchicine were added at growth start, at different final concentrations, up to 2.5-3 g/L. Frozen cultures of *B. megaterium* that were conserved in liquid nitrogen as frozen suspensions in Luria-Bertani (LB) medium added with 20-50% glycerol, were utilized for the inoculum of seed cultures in 1,000 mL Erlenmeyer flasks, washing with water (2.0 L) at a flow rate of 1 BV/h, the product was washed, with methanol (2.0 L) at a flow rate of 1 BV/h. The methanol was concentrated to dryness under vacuum, yielding 47.0 g of solid. The latter was dissolved in 50% v/v methanol (230 mL) and extracted with dichloromethane (10 x 60 mL). The hydroalcoholic phase was concentrated to dryness, providing 24.2 g of solid. The latter was dissolved in a 1:1 ethanol-dichloromethane mixture (470 mL). The solution was clarified on a pad of silica gel (5.0 g), which was then washed with the same solvent mixture (100 mL).

Dichloromethane was removed by evaporation; the resulting solution was concentrated to 140 mL and left to crystallize overnight at 25 °C. The solid was filtered and dissolved in a 1:1 ethanol-chloroform mixture (470 mL). The solution was clarified on a pad of silica gel (5.0 g), which was then washed with the same solvent mixture (100 mL). Chloroform was removed by evaporation; the resulting solution was concentrated to 140 mL and left to crystallize overnight at 25 °C. Thiocolchicoside was obtained as a yellow solid (18.4 g) [13]. The physical and chemical properties of Thiocolchicoside are presented in Table 2. The details of the stability and degradation profile of Thiocolchicoside are presented in Table 3.

**Table 2:** Physiochemical properties of Thiocolchicoside mg/ml: milligram per millilitre, g/mol: grams per mole, µm: micrometers, pKa: negative logarithm of the acid dissociation constant

Sr. No.	Property	Comment
1	Formula [10]	C_27_H_33_NO_10_S
2	Solubility [14]	High water solubility (16.1 mg/ml)
3	Permeability [14]	Octanol has a low partition coefficient with water. (log P = -0.34) and high molecular weight (563.62 g/mol), so less lipid permeable.
4	Physical state [15-16]	Yellow crystalline powder that dissolves in water and DMSO.
5	Polarity [14]	Polar molecule
6	Particle size [17]	1-1000 µm
7	Melting point [18]	190-198 °C
8	Boiling point [18]	929.624 °C
9	Ionisation [14]	The pKa value is 12.74.
10	Molecular weight [14]	563 g/mol

**Table 3:** Stability and degradation profile of Thiocolchicoside HCl: hydrochloric acid, NaOH: sodium hydroxide, H_2_O_2_: hydrogen peroxide

Degradation type	Conditions	Observations	Retention factor (Rf) values
General stability	60 days at standard storage [19]	Less than 0.5% degradation; indicates good stability [18]	—
Acidic degradation [20]	1.0 M HCl at 60°C, 30 minutes [20]	Unstable; two degradation peaks formed [20]	0.33, 0.71 (Degraded products) [20]
Basic degradation [20]	0.5 M NaOH at 60°C, 30 minutes [20]	Unstable; one degradation peak observed [20]	0.60 (Parent), 0.72 (Degraded product) [20]
Oxidative degradation (1%) [20]	1% v/v H₂O₂ [20]	Three degradation peaks formed; parent compound remains [20]	0.38, 0.46, 0.70 (Degraded), 0.60 (Parent) [20]
Oxidative degradation (3%) [20]	3% v/v H₂O₂ [20]	Complete decline was seen, with two major peaks and one minor peak. [20]	0.58, 0.64, 0.70 (Degraded products) [20]
Photolytic degradation [20]	Direct sunlight for eight hours [20]	Degradation occurs in methanolic solution after light exposure [20]	Not specified
Degradation susceptibility	Summary of degradation order	0.1N NaOH > Photolysis > 3% H₂O₂ > 0.1N HCl [21]	—

Pharmacokinetics

Absorption

The absorption of drugs is contingent upon their physicochemical characteristics and the method of delivery. Lipid-soluble drugs often traverse biological membranes more efficiently via passive diffusion, while water-soluble drugs may necessitate carrier-mediated transport or be confined to paracellular routes. Thus, absorption rates fluctuate and cannot be generalized merely based on solubility. Drugs in aqueous solutions are absorbed more quickly as they mix with the aqueous phase at the absorption site more easily than those in oily solutions [22]. Thiocolchicoside has a low octanol/water partition coefficient (log P = -0.34), a fairly high molecular weight (563 g/mol), and a reasonably high-water solubility (16.1 mg/ml). It is a Biopharmaceutics Classification System (BCS) class III [14] drug having high solubility and low permeability. Following intramuscular (IM) injection, the maximum plasma concentration (C max) of Thiocolchicoside occurs in 30 minutes and reaches values of 113 ng/mL after a 4 mg dose and 175 mg/mL after an 8 mg dose [23].

Distribution

A pharmacokinetic measure called the volume of distribution (Vd) indicates the probability of a drug either staying in the plasma or dispersing to different tissue compartments. A drug with a higher Vd gets diffused into the tissues, and a higher dose is needed to achieve the desired plasma concentration. Thiocolchicoside, being a hydrophilic drug, has a low Vd and is more likely to stay in the bloodstream and is less likely to penetrate through lipid bilayers [24]. Regarding its plasma protein binding, studies using centrifugation and equilibrium dialysis have demonstrated that Thiocolchicoside and its derivative bind to human serum and pure human protein at 38.90 C and 12.80 C±5.3% with albumin [23]. The apparent volume of distribution of Thiocolchicoside is estimated to be approximately 42.7 L, following an IM dose of 8 mg [23].

Metabolism

It is a process of chemical alteration of a drug that occurs in the liver, kidneys, intestine, lungs, and plasma. Drug metabolism enhances polarity, hence promoting renal excretion and preventing them from reabsorbing into renal tubules, promoting their excretion. However, for water-soluble drugs, they are excreted unchanged without chemical alteration [24]. Following oral ingestion, Thiocolchicoside is quickly absorbed and breaks down into three primary metabolites. First, to 3-demethylcolchicine (an inactive metabolite) in the intestine. In the body, this compound is broken down even more by either demethylation to di-demethylcolchicine (an inactive metabolite) or conjugation to 3-O-glucurono-demethylcolchicine (an active metabolite) [25].

Elimination

Following IM administration, the plasma clearance is 19.2 L/h and the apparent half-life of Thiocolchicoside is 1.5 hours. Urinary excretion accounts for just 20% of total radioactivity after oral treatment, with feces accounting for 79%. In either urine or feces, Thiocolchicoside is eliminated unmodified. SL18.0740 and SL59.0955 are present in both urine and stool; however, di-demethyl-thiocolchicine is observed just in stool. After taking Thiocolchicoside by mouth, the metabolite SL18.0740 is removed from the body with a half-life of 3.2 to seven hours, whereas the metabolite SL59.0955 has a half-life of 0.8 hours [25].

Pharmacodynamics

*Analgesic* *Activity*

Thiocolchicoside has analgesic effects predominantly by modulating inhibitory neurotransmission, functioning as an antagonist at GABA-A and glycine receptors in the brain and spinal cord [26].

Anti-inflammatory Activity

Tumor necrosis factor (TNF) induces nuclear factor kappa B (NF-κB) binding sites in cyclooxygenase-2 (COX-2). Thiocolchicoside suppresses NF-κB-regulated gene products [7] and downregulates NF-κB, leading to anti-inflammatory effects. Cytokines, including TNF, carcinogens, tobacco smoke, environmental contaminants, ionizing radiation, and stress, can activate NF-κB. Thiocolchicoside reduces TNF and COX-2 activity in a dose-dependent manner [7]. Thiocolchicoside lowers NF-κB, which binds to the COX-2 promoter and regulates gene expression.

Skeletal Muscle Relaxant Activity

Skeletal muscle relaxant action is mediated by its strong and specific affinity for GABA-A receptors. It increases GABA-mediated inhibitory neurotransmission by binding to these central nervous system receptors. This activates the GABAergic inhibitory pathways that lower motor neuron activity and produce efficient centrally mediated muscle relaxation without noticeable drowsiness by significantly reducing muscular contractures and stiffness. By hyperpolarizing post-synaptic neurons, glycine, an inhibitory neurotransmitter found in the brainstem and spinal cord, modifies motor and sensory transmission. Thiocolchicoside increases inhibitory synaptic signalling by its simultaneous action on GABA-A and glycine receptors [27], which results in decreased muscular tone, skeletal muscle relaxation, and relief from painful spasms linked to neurological and musculoskeletal problems.

Proconvulsant Activity

Thiocolchicoside interacts with glycine receptors sensitive to strychnine, and GABA type A receptors [28]. Glycine receptors, mostly situated in the brainstem and spinal cord, often facilitate inhibitory neurotransmission and aid in muscle relaxation. Thiocolchicoside diminishes inhibitory tone by obstructing the allosteric activation of these receptors. Conversely, specific subtypes of GABA type A receptors in the cerebral cortex seem to be more significantly impacted, resulting in cortical hyperexcitability and convulsive activity [26]. The prevalence of GABA type A receptors in the brain relative to glycine receptors elucidates the ability of Thiocolchicoside to induce seizures, as emphasized in pharmacological safety investigations [29] and regulatory hurdles.

*Anticancer* *Activity*

Thiocolchicoside may serve as an anticancer agent by reducing cell viability through the induction of apoptosis. By inducing the cleavage of poly (ADP‑ribose) polymerase (PARP) and caspase‑3 [7], it facilitates programmed cell death, a conclusion further supported by a recent experimental investigation in oral cancer cell lines, whereby thiocolchicoside nanogel exhibited pro-apoptotic and anticancer properties [6]. It decreases cell viability by the activation of many molecular mechanisms, such as the induction of apoptosis, inhibition of cell survival proteins, suppression of NF‑κB signaling, reduction of cell proliferation indicators, and downregulation of anti-apoptotic pathways, as shown below.

Induction of Apoptosis

By stimulating the cleavage of poly (ADP-ribose) polymerase (PARP) and caspase-3 [7], Thiocolchicoside promotes apoptosis or programmed cell death. Cell death is caused by the controlled breakdown of nuclear DNA, cytoskeletal proteins, and mitochondrial integrity, leading to apoptotic body formation.

Cell Survival Protein Suppression

It reduces the expression of cell survival proteins, such as cIAP-1, cIAP-2, bcl-xL, MCL-1, XIAP, Bcl-2, and cFLIP [7]. Suppression of these proteins increases the likelihood of programmed cell death because they typically aid cells in avoiding apoptosis.

Inhibition of the NF-κB Pathway

Thiocolchicoside inhibits the NF‑κB pathway, which regulates inflammation, cell survival, and proliferation. Specifically, it downregulates NF‑κB‑dependent anti‑apoptotic and survival genes, including Bcl‑2, Bcl‑xL, XIAP, Mcl‑1, cIAP‑1, cIAP‑2, and cFLIP [7]. Suppression of these gene products enhances apoptosis and reduces tumor cell proliferation.

Inhibition of Cell Proliferation Biomarkers

Thiocolchicoside attenuates the phosphorylation of PI3K (p85 subunit), GSK3β, and c-Myc, which are essential regulators of cellular survival and proliferation. It has anti-cancer properties that increase with dose [30]. Thiocolchicoside has demonstrated selective cytotoxicity towards malignant cells in experimental settings, with minimal evidence of impact on normal cells [31]. Nonetheless, additional research is necessary to validate its selectivity among other cell types.

Suppression of Antiapoptotic Proteins

Thiocolchicoside inhibits cell proliferation proteins such as PCNA, Ki67, and MCM-2. At higher doses, it also lowers the levels of the antiapoptotic proteins Bcl-2, XIAP, Mcl-1, Bcl-xL, cIAP-1, cIAP-2, and cFlip [7, 30]. Thiocolchicoside, on the other hand, activates caspase-3 and PARP, indicating that it has proapoptotic activity. Thiocolchicoside stops the phosphorylation of the p85 subunit of PI3K, GSK3β, and c-myc. In this context, Thiocolchicoside is believed to inhibit the dissemination of cancer cells by hindering cell division and commencing apoptosis.

Inhibition of TNF and NF-ĸB Activation

NF-κB, a transcription factor, governs cellular apoptosis and proliferation. Through the utilization of the electrophoretic mobility shift assay (EMSA), it has been proven that the inhibition of TNF-induced NF-κB activation occurs in a dose- and time-dependent way by Thiocolchicoside [7]. Thiocolchicoside is incapable of directly binding to DNA. Thiocolchicoside indirectly suppresses NF-κB activation rather than directly.

Suppression of NF-ĸB Activation

Numerous carcinogens, tumor promoters, and inflammatory compounds, including lipopolysaccharide (LPS), phorbol 12-myristate 13-acetate (PMA), and osteoarthritis (OA), activate NF-κB through potentially distinct methods. All of these substances stimulated NF-κB, according to the EMSA, but Thiocolchicoside inhibits their activation [7].

Inhibition of TNF-Dependent Inhibitor of Kappa B Alpha (IκBα) Phosphorylation, Ubiquitination, and Degradation

IκBα is broken down by proteases before NF-κB is translocated to the nucleus. According to the EMSA, NF-κB gets activated as TNF duration increases, and this activation is significantly reduced by the pretreatment of Thiocolchicoside. Thicolchicoside also reduces TNF-induced IκBα degradation, according to the Western blot test. Alongside IκBα degradation, Thiocolchicoside also prevented IκBα phosphorylation. Thiocolchicoside inhibited TNF-induced phosphorylation, IκBα degradation, and NF-κB activation [7]. Thiocolchicoside reduces IκBα ubiquitination and TNF-induced IκBα phosphorylation, leading to an anti-cancer action, as shown by a Western blot study employing an antibody that binds to the phosphorylation of the IκBα derivative.

Inhibition of TNF-Initiated Activation of IκB Kinase (IKK)

Phosphorylation of IκBα by TNF necessitates IKK, which activates NF-κB and facilitates its movement from the cytoplasm to the nucleus, thereby promoting the transcription of target genes and contributing to cancer development. Thiocolchicoside stops TNF from activating IKK, and TNF activates IKK in a time-dependent way [7].

Inhibition of Nuclear Translocation of p65

P65, a component of NF-κB, contains nuclear translocation signals. Thiocolchicoside inhibits p65 shifting from the cytoplasm to the nucleus. Thiocolchicoside also inhibits p65 phosphorylation at Serine 536 (Ser536) [7].

Suppression of NF-κB-Dependent Genes Induced by TNF

Thiocolchicoside stops NF-κB activation and the expression of the secreted embryonic alkaline phosphatase (SEAP) gene in a dose-dependent way [7].

TNF Receptor and NF-κB Gene Expression Suppression

TNF activates NF-κB by interacting with TNFR1, TRADD, TRAF2, NIK, and IKKβ, leading to the phosphorylation of IκBα. TNF activates NF-κB by interacting with NIK, TRADD, TRAF2, TNF receptor 1, and IKKβ, which phosphorylates IκBα. Plasmids showing TNFR1, TRADD, TRAF2, NIK, IKKβ, and p65 were put into cells. Plasmid-transfected cells expressed NF-κB-controlled reporter genes [7]. Thiocolchicoside suppresses reporter expression caused by any of the plasmids.

Inhibition of TNF-Induced Upregulated COX-2 Activity

Thiocolchicoside prevents the dose-dependent reduction of TNF-induced COX-2 activity [7]. Thiocolchicoside prevents NF-κB from binding to COX, which suppresses gene expression regulated by the protein.

Antimicrobial Activity

Thiocolchicoside is posited to function as an antimicrobial agent via two mechanisms: (i) the creation of ion pores in lipid bilayer membranes, which undermines their electrical insulating properties and results in modified ion conductance [4‑5], and (ii) the inhibition of protein and nucleic acid synthesis, consequently diminishing microbial replication [4‑5]. Research on membranes indicates that the pores created are of the toroidal configuration, wherein lipid monolayers curve to converge around the pore aperture, thereby weakening the bilayer. Their synergistic actions offer a credible molecular foundation for their antibacterial efficacy.

Antianxiety Activity

Thiocolchicoside is hypothesized to act as an anti-anxiety drug by the following mechanism.

Thiocolchicoside demonstrates a high affinity for glycinergic and inhibitory GABA receptors. Its myorelaxant effect may be due to its agonistic action on strychnine-sensitive spinal receptors. Thiocolchicoside is thought to be used as a monotherapy for anxiety disorders because of its affinity for GABA receptors [32]. It also potentiates the effect of diazepam.

Myelosuppressive Activity

Thiocolchicoside, due to its structural interaction with colchicine, may have an indirect myelosuppressive effect; however, the present evidence is hypothetical and requires more experimental validation. It may act as a myelosuppressive by

Bone Marrow Suppression

Colchicine can directly affect the bone marrow, which leads to a decrease in the formation of red blood cells, white blood cells, and platelets [8].

Inhibition of Mitosis

Colchicine obstructs mitotic spindle assembly by binding to tubulin and inhibiting microtubule polymerization, resulting in cell arrest in metaphase and inducing death [33-34]. Thiocolchicoside, a semi-synthetic derivative, is posited to influence microtubule dynamics similarly, resulting in diminished proliferation of bone marrow cells and myelosuppression.

Drug interactions

Colchicine increases the risk of myelosuppression in patients who already have renal impairment or who are taking drugs that block cytochrome P450 3A4 (CYP3A4) or P-glycoprotein (P-gp) [8].

Toxicity

Colchicine can have harmful effects on the bone marrow that can worsen with overdose or chronic usage, resulting in severe cytopenias (decreases in blood cell counts). Due to its structural similarity to colchicine, Thiocolchicoside may indirectly produce myelosuppressive effects; nevertheless, existing evidence is speculative and necessitates further validation. 

Mechanism of action

It inhibits the glycine receptor and functions as a competitive antagonist of the GABA type A receptor. To a much lesser extent, it also acts on nicotinic acetylcholine receptors. It reduces motor neuron excitability, leading to skeletal muscle relaxation by enhancing inhibitory neurotransmission in the spinal cord. It does not interfere with cholinergic transmission, unlike other neuromuscular blocking agents, preventing flaccid paralysis [3]. 

Clinical uses

Central Nervous System

*I*t can be used for the treatment of muscle spasms that are linked to neurological disorders, like multiple sclerosis, cerebral palsy, and stroke [35].

Local Tissue Injuries or Muscle Strain [35] and Myofascial Pain Syndrome

The ointment appears to be an effective alternative for people who are unable to get injections [35].

Oral Submucosal Fibrosis

The pilot study evaluated the use of this therapy in conjunction with the use of drugs that relax muscles [35].

Bone Loss

Thiocolchicoside inhibits osteoclastogenesis produced by receptor activator of nuclear kappa-B ligand in breast cancer and multiple myeloma cells via inhibiting pathways of inflammation [35].

Thiocolchicoside has been studied for its potential as an analgesic in myofascial pain syndrome [36], dental pain management [37], and low back pain associated with spasms [38-39].

Proposed uses

The proposed uses are antimicrobial by forming ion pores in between the membrane, inhibiting protein synthesis and nucleic acid synthesis [4-5], and anti-cancer by modulating NF-κB regulating proteins [6-7].

Various dosage forms of Thiocolchicoside

Tablets

Thiocolchicoside is available in tablet form as 4 mg, 8 mg, and 16 mg. Fixed-dose combinations for 4 mg preparations are available: Etoricoxib 60 mg + Thiocolchicoside 4 mg, Aceclofenac 100 mg + Thiocolchicoside 4 mg, Ketoprofen 50 mg + Thiocolchicoside 4 mg. Diclofenac 50 mg + Thiocolchicoside 4 mg + paracetamol 325 mg, Diclofenac 50 mg + Thiocolchicoside 4 mg. In 8 mg preparations, examples of fixed-dose combinations are available: Thiocolchicoside 8 mg + Aceclofenac 100 mg + paracetamol 325 mg, Thiocolchicoside 8 mg + Etoricoxib 60 mg, Thiocolchicoside 8 mg + Aceclofenac 100 mg, diclofenac 50 mg + Thiocolchicoside 8 mg. In the 16 mg preparation, the available is Aceclofenac 200 mg + Thiocolchicoside 16 mg [40].

Parenteral

IM injections of Thiocolchicoside in doses of 2 mg and 4 mg are available. They are also available in fixed-dose combinations: Thiocolchicoside 2 mg + diclofenac 37.5 mg injection. For 4 mg, it is available as a 4 mg/2 ml ampoule [40].

Ointment

Thiocolchicoside can be used to treat myofascial pain syndrome. Thiocolchicoside ointment may be regarded as a safe and efficacious dosage alternative as an alternative to injections for patients or other forms of administration [36].

Gel/Transdermal

Chitosan/hyaluronan transdermal films were developed to increase bioavailability. This technique evades the digestion process, and simultaneously, it can give an anticipated and broadened length of action. This novel mechanical stage for transdermal medication transportation and its outcomes highlight the significance of chitosan/hyaluronan polyelectrolyte forms as new materials for easy administration [41-42].

 *Foam*

The innovative Thiocolchicoside foam formulation contains 0.25% of Thiocolchicoside in addition to the following excipients: polysorbate 80 (4%), propylene glycol (4%), alcohol (ethanol; 20%), propylene glycol diperlargonate (1%), benzyl alcohol (1%), sodium phosphate monobasic monohydrate (0.83%), sodium phosphate dibasic dodecahydrate (0.23%), and nerolene lavender (0.20%). Propane and butane gases were used as gas propellants, and a formulation was made to dispense foam [43].

Nanoemulsion Enriched With Omega-3 Fatty Acids

The nanoemulsions increase the transdermal delivery of Thiocolchicoside. Overall, the nanoemulsions were found to be effective in transdermal administration of Thiocolchicoside with anti-inflammatory effects [44].

Advantages of gel over the tablet form 

Table 4 shows the advantages of gel over the tablet form [45].

**Table 4:** Advantages of gel over tablet form NSAIDs: non-steroidal anti-inflammatory drugs

Feature	Gel formulation	Tablet form
Bioavailability	Bypasses liver metabolism, enhancing systemic drug levels	Undergoes first-pass liver metabolism, which can reduce effectiveness
Targeted delivery	Enables localized treatment by direct skin application at the site of pain	Distributes systemically with minimal localization
Onset of action	Provides quicker relief due to systemic absorption [27]	Slower effect onset owing to absorption through the digestive tract
Gastrointestinal tolerance	Avoids irritation and food-related interactions	May cause GI disturbances and interact with food
Patient compliance	Suitable for those who cannot or prefer not to take oral medications	Requires swallowing, which may be difficult for some patients
Controlled release potential	Could be formulated for extended or release of drug using polymers	Lacks mechanisms for sustained release
Systemic side effects	Its topical dosage form lowers risk of adverse effects [27]	Higher systemic drug levels may lead to increased side effects
Formulation flexibility	Allows inclusion of other active agents (e.g., NSAIDs) for enhanced therapeutic effect	Limited ability to combine multiple drugs in a single formulation

Recent research

Antimicrobial Activity

An in vitro study done in 2023 by Das et al. investigated the antimicrobial efficacy of Thiocolchicoside using the broth dilution procedure to find the minimum inhibitory concentration (MIC) against *Staphylococcus aureus, Escherichia coli, Pseudomonas aeruginosa*, and *Proteus mirabilis*, with values spanning from 0.48 µg/ml to 1000 µg/ml. Bacterial strains exhibited diminished susceptibility to the myorelaxant activity, with MICs of 500 μg/mL for *E. coli* and 1000 μg/mL for* P. aeruginosa* [46]. A similar in vitro study was carried out in 2021 by Md. Ashrafuzzaman, who compared Thiocolchicoside with gramicidin-S for lipid bilayer membrane disrupting property using the electrophysiology record of membrane current (ERMC) method, in which an artificial lipid bilayer membrane was created using a mixture of phosphoethanolamine (POPE)/phosphatidylserine (POPS)/ phosphatidylcholine (POPC) (5:3:2, v/v/v)/n-decane. Gramicidin-S was used in nano-molar concentrations. The results showed Thiocolchicoside-induced toroidal-type ion pores in lipid bilayer membrane after a transmembrane current of 100 mV was applied [4]. Moreover, in 2023, Ameena M et al. explored anti-inflammatory, antimicrobial, antioxidant, and cytotoxic effects using chitosan thiocolchicoside-lauric acid nanogel (CTLA) against *Streptococcus mutans, S. aureus, Pseudomonas aeruginosa, Lactobacillus, *and *Candida albicans* using the well diffusion method. The results showed that the nanogel demonstrated a 20 mm zone of inhibition against *S. mutans* and a 22 mm zone of inhibition against *S. aureus* when 100 µg/mL of CTLA nanogel was diluted, predicting the role of Thiocolchicoside for antimicrobial activity [5]. Nonetheless, its clinical relevance remains unsubstantiated, as existing evidence is confined to in vitro investigations.

Anticancer Activity

In vitro studies carried out in 2010 by Simone Reuter et al. investigated the anti-cancer effects of Thiocolchicoside on the NF-κB cell signaling system and on cellular responses mediated by NF-κB on cell lines, namely, U266, RPMI-8226, Jurkat, MM.1S, Caco-2, HT-29, A293, HCT116, MCF-7, MCF-10A, KBM5, and SCC4 cells. The results showed that Thiocolchicoside suppresses both the activity of NF-κB reporter and the promoter of cyclooxygenase-2 [7]. A similar type of study was carried out in 2024 by Mustafa et al. on a cell line using 0.05 gm Thiocolchicoside and 0.5 gm lauric acid loaded chitosan nanogel against the KB1 cell lines by the MTT assay. The results showed that the nanogel possessed maximum cytotoxicity when compared to the control [6]. In silico molecular docking study on TRAF6-RAN and anti-proliferative study on triple-negative breast cancer (TNBC) for the TNBS cell line was conducted by Shreya Medhi et al. in the year 2021, which showed an energy binding score of -5.11 kcal/mol and decrease in cell viability (95.93%, 62.33%, 55.56%, 53.66%, 44.17%, and 39.84%) when it was treated with Thiocolchicoside using different concentrations (15.625 µM, 31.25 µM, 62.5 µM, 125 µM, 250 µM, and 500 µM) [47]. A similar type of study was carried out in 2024 for molecular docking by Mustafa et al. to evaluate the interaction of Thiocolchicoside with therapeutic cancer targets, including PAK4, wild-type TP53, and TP53 mutants (Y220C, R175H, and R248Q) for oral cancer cell lines KB-1. The results showed that Thiocolchicoside demonstrated strong binding affinities with all the tested targets, particularly PAK4 (-7.28 kcal/mol), indicating its potential as a therapeutic agent. Nonetheless, docking studies alone are unable to determine therapeutic efficacy, as they neglect pharmacokinetics, metabolism, and in vivo biological intricacies. Additional experimental validation is necessary. The interactions with wild-type TP53 and mutants suggest that Thiocolchicoside has the potential to stabilize the function of the protein or re-establish its tumor-suppressor activity [47].

Drug repurposing

Drug repurposing, or drug repositioning, refers to the identification of novel applications for drugs that extend beyond their established therapeutic indications. Typically, approved medications or medications that were withdrawn from clinical trials for reasons unrelated to safety are the drug candidates that are selected. Clinical trials for these drugs are carried out more quickly, more affordably, and with lower risk than traditional drug development techniques. This is feasible as these drugs' safety characteristics are already established. The established clinical use of Thiocolchicoside as a muscle relaxant offers a basis for repositioning tactics, but EMA limits underlying serious safety concerns. Consideration of repositioning strategies is facilitated by the established clinical use of Thiocolchicoside as a muscle relaxant. However, safety concerns, including genotoxicity, must be carefully evaluated. The capacity of small compounds to target specific proteins in cells justifies therapeutic repositioning. Thiocolchicoside is a prime example of this, as it has the potential to reposition itself by antagonizing glycine and GABA-A receptors, while its activity at nicotinic receptors is attenuated [12,26-27]. We can monitor particular pathways implicated in the pathophysiology of the identified disease in this way. The same chemical in this instance may target distinct pathways that may be unrelated to one another, yet are implicated in the initiation or advancement of cancer. This polypharmacology is evident in the reported anticancer activity, antimicrobial activity, and inhibition of NF-κB in vitro of Thiocolchicoside [4-7,48].

"Polypharmacology" is the term for this idea, which deviates slightly from the conventional strategy of finding a single medicine for a single target to achieve high selectivity and improved efficacy. An example of this notion is the polypharmacological profile of Thiocolchicoside acting on several receptor systems [1,26-27]. In this instance, the primary goals are also to lessen toxicity and stop the patient from developing any type of drug resistance. Nevertheless, the genotoxic metabolites and the epileptogenic potential of Thiocolchicoside are drawbacks that must be strictly evaluated [9,28]. Drug repurposing has drawn a lot of interest recently from academic institutions and pharmaceutical corporations in an effort to address the fundamental flaws in de novo drug discovery. Thiocolchicoside, with its well-known pharmacological data and clinical application, is such a candidate, but its repositioning potential has to be balanced with regulatory constraints like EMA and unresolved safety risks. Repositioning pharmaceuticals offers several benefits over de novo drug discovery since the repositioned drug has previously undergone numerous rounds of clinical trials and toxicity testing, establishing its safety and lowering the possibility of failure from unfavourable toxicology profiles. A quicker and less likely path to drug development is to begin with an established drug or a molecule with a wealth of clinical data [46-48]. It showed the desired effect, paving a way for an alternative where resistance to the therapy looms at large, leading to treatment failure.

Adverse drug reactions

Thiocolchicoside has been linked to the number of adverse reactions, like seizures [35], diarrhea, gastralgia, nausea and vomiting [35], pruritis, urticaria, angioedema [35], anaphylactic shock following muscular injections, vasovagal syncope [35], pregnancy-related conditions (e.g., miscarriages, still birth, preterm birth, low birth weight, and elective terminations) [35], major congenital malformations (e.g., cleft lip with palate and bilateral hip dislocation) [35], and minor congenital malformations (bilateral vesicoureteral reflux, genotoxic M2 metabolite (SL59.0955) produced by the deglycosylation of Thiocolchicoside that induces aneuploidy). This molecule inhibits spindle function and induces chromosomal disaggregation, leading to aneuploidy in dividing cells. These molecular insights justify the restriction of Thiocolchicoside usage by EMA due to dose-dependent genotoxicity and safety concerns, such as hepatotoxicity [35], hearing loss [49], intrauterine growth retardation, and patent foramen ovale [50].

Contraindications

It should not be used when pregnant or lactating due to genotoxic metabolites and those tending to have a history of epilepsy [28,35]. Patients must be adequately informed about any risks associated with pregnancy and the implementation of appropriate contraceptive methods. It should not be administered to pediatric patients [35]. It is avoided in persons who become hypersensitive to it [51]. Children under the age of 16 should not take the drug [35].

Safety concerns

De-glycosylation of Thiocolchicoside results in the genotoxic metabolite M2 (SL59.0955) that induces aneuploidy. M2 inhibits the activity of the spindle and causes chromosomal disaggregation in dividing cells. Due to the dose-dependent genotoxic effects, the EMA restricted the use of Thiocolchicoside and contraindicated its use in pediatric patients, pregnant women, lactating women, and those with a history of seizures [28,35]. Although Thiocolchicoside is an efficient muscle relaxant, its therapeutic use must be evaluated against the potential risks of genotoxicity, aneuploidy, and regulatory constraints, highlighting the necessity for a definitive risk-benefit analysis. 

Discussion

Global Burden and Context

Globally, an estimated 1.57 billion people had hearing loss in 2019, with 430 million requiring rehabilitation. By 2050, approximately 2.5 billion individuals are expected to be affected, with over 700 million requiring rehabilitation [52]. This epidemiological burden emphasizes the significance of investigating innovative treatment drugs like Thiocolchicoside.

Clinical Evidence and Therapeutic Significance

Preclinical investigations indicate that Thiocolchicoside inhibits NF‑κB signaling and downregulates gene products associated with protein synthesis, hence supporting its potential anticancer and anti‑inflammatory properties [7]. Randomized controlled trials and comprehensive reviews have established efficacy in managing musculoskeletal pain [53]; however, regulatory bodies like the European Medicines Agency have limited usage due to concerns over genotoxicity and aneuploidy risks. Comparative investigations indicate that Thiocolchicoside offers muscular relaxation comparable to benzodiazepines and other centrally acting drugs; nonetheless, its unique mechanism (antagonism of GABA-A and glycine receptors, inhibition of NF-κB) necessitates a thorough risk-benefit evaluation. Clinical guidelines underscore the prudent application, especially in demographics susceptible to seizures or reproductive harm.

Comparative Analysis of Alternative Muscle Relaxants

In contrast to drugs such as tizanidine or baclofen, Thiocolchicoside provides peripheral muscular relaxation with little sedation, although it has distinct safety issues. The incorporation of clinical data with chemical taxonomy provides a comprehensive viewpoint that is consistent with therapeutic practice.

## Conclusions

Thiocolchicoside is a clinically validated muscle relaxant having a well-documented function in the treatment of musculoskeletal ailments. In addition to its conventional applications, recent research underscores its potential antibacterial and anticancer characteristics, facilitated by mechanisms including apoptosis induction, suppression of cell survival proteins, inhibition of NF‑κB signaling, and reduction of cell proliferation indicators. These data indicate that Thiocolchicoside may be a viable candidate for drug repurposing in oncology and infectious disease treatment. Nevertheless, the majority of existing data are from in vitro and preclinical investigations, and their use in clinical practice remains ambiguous. Concerns about genotoxicity, myelosuppression, and dose-dependent toxicity require careful evaluation of its therapeutic potential. Future research must focus on meticulously constructed in vivo investigations and clinical trials to confirm efficacy, refine dosing regimens, and guarantee safety. Furthermore, innovative drug delivery technologies like nanogels and customized formulations may improve bioavailability while reducing unwanted effects. Thiocolchicoside is a versatile pharmacological substance with broadening therapeutic uses. Its dual antibacterial and anticancer characteristics, along with recognized muscle relaxant effects, highlight its significance as a primary candidate for further research. Thorough clinical validation will be crucial to ascertain if these first discoveries can be converted into significant therapeutic uses.
